# Carbon Dioxide and Water Activation by Niobium Trioxide Anions in the Gas Phase

**DOI:** 10.1021/acs.jpca.3c01394

**Published:** 2023-04-11

**Authors:** Magdalena Salzburger, Rizalina T. Saragi, Frank J. Wensink, Ethan M. Cunningham, Martin K. Beyer, Joost M. Bakker, Milan Ončák, Christian van der Linde

**Affiliations:** † Institut für Ionenphysik und Angewandte Physik, Universität Innsbruck, Technikerstraße 25, 6020 Innsbruck, Austria; ‡ Radboud University, Institute for Molecules and Materials, FELIX Laboratory, Toernooiveld 7, 6525 ED Nijmegen, The Netherlands

## Abstract

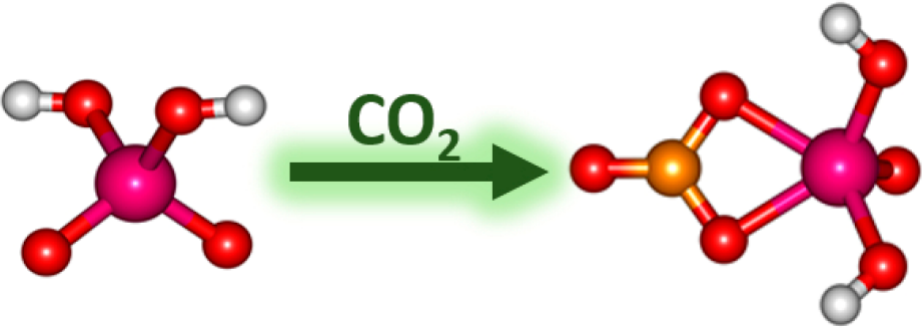

Transition metals are important in various industrial applications including catalysis. Due to the current concentration of CO_2_ in the atmosphere, various ways for its capture and utilization are investigated. Here, we study the activation of CO_2_ and H_2_O at [NbO_3_]^−^ in the gas phase using a combination of infrared multiple photon dissociation spectroscopy and density functional theory calculations. In the experiments, Fourier-transform ion cyclotron resonance mass spectrometry is combined with tunable IR laser light provided by the intracavity free-electron laser FELICE or optical parametric oscillator-based table-top laser systems. We present spectra of [NbO_3_]^−^, [NbO_2_(OH)_2_]^−^, [NbO_2_(OH)_2_]^−^(H_2_O) and [NbO(OH)_2_(CO_3_)]^−^ in the 240–4000 cm^–1^ range. The measured spectra and observed dissociation channels together with quantum chemical calculations confirm that upon interaction with a water molecule, [NbO_3_]^−^ is transformed to [NbO_2_(OH)_2_]^−^ via a barrierless reaction. Reaction of this product with CO_2_ leads to [NbO(OH)_2_(CO_3_)]^−^ with the formation of a [CO_3_] moiety.

## Introduction

Transition metals and their oxides are important in materials science and industrial applications.^1−4^ Some of them, such as niobium oxides, are used as catalysts.^5−10^ Since the 1990s, several studies have been published investigating the chemical properties of niobium oxides, with a focus on ionic clusters, particles with a countable number of atoms that can sensitively be probed using mass spectrometry. The formation and structures of niobium oxide cluster ions themselves have been studied using ion mobility and collision-induced dissociation mass spectrometry and infrared spectroscopy.^11−14^ Their reactivity was explored by studying the reactions with *n*-butane,^15^ H_2_,^16^ H_2_O,^17,18^ methanol and ethanol,^19^ including a series of oxidation and reduction reactions of [NbO_3_]^−^.^20^ Deng et al. demonstrated that some niobium oxides adsorb small hydrocarbons at near-thermal energies and abstract an oxygen atom from oxygen-containing molecules.^11^ Spectroscopic studies are limited to infrared spectra of several niobium oxide cations,^13,14^ photoelectron spectra of [NbO_3_]^−^ and [Nb_3_O_2_]^−^,^21^ and matrix isolation infrared spectra of niobium oxide anions, including [NbO_3_]^−^.^22^ Computationally, Su et al. investigated the oxidation states of [NbO_3_]^−^ using relativistic quantum chemistry.^23^

Reaction products involving niobium or niobium oxide cluster ions have also characterized. The complex of Nb^+^ with a single water molecule has been investigated by infrared photodissociation spectroscopy using the rare-gas tagging method.^24^ The reaction of [NbO_3_]^−^ with one water molecule was explored by Sigsworth et al. in an experimental study with a fast-flow reactor, showing that [NbO_3_]^−^ reacts by forming [NbO_2_(OH)_2_]^−^ with a rate coefficient of 3 × 10^–9^ cm^3^ s^–1^.^17^ Sambrano et al. calculated the reaction pathway to form [NbO_2_(OH)_2_]^−^, finding no barrier for this reaction.^18^ To the best of our knowledge, no spectroscopic characterization of hydration products of [NbO_3_]^−^ has been done.

Because niobium oxides can activate carbon dioxide,^16^ they are potentially relevant for mitigation of the increasing atmospheric CO_2_ levels. CO_2_ activation has been investigated already in detail on many systems, for example, on atomic metal anions^25,26^ and cations^27−30^ including Nb^+^,^31^ and the general picture emerges that it is easier to activate CO_2_ by anions than by cations or neutrals. This is attributed to the additional electron density of the anions, which can be donated into the CO_2_ antibonding 2π_u_ lowest unoccupied molecular orbital, weakening the O–CO bond.^32,33^ Additionally, early transition metals are better suited for CO_2_ activation than late transition metals because of their higher oxophilicity, promoting a possible oxygen atom transfer from CO_2_.^34^ Apart from atomic ions, CO_2_ activation was also studied in clusters of selected metals.^35−42^ In line with the above, for cationic Cu_*n*_^+^ clusters only nonactivated adsorption was observed, substantiated by large calculated reaction barriers hindering the activation.^41^ For anionic clusters, size-selective CO_2_ activation was observed for Pt_*n*_^–^, Co_*n*_^–^ and C-doped Cu_*n*_^–^ clusters,^36,39,40^ whereas for neutral Cu clusters, doping with 3d elements was computationally shown to allow control over activation.^40,42^ CO_2_ activation has further been studied, for example, on hydrogenated metals,^43−45^ metal carbide clusters,^46,47^ and metal oxide species,^29,48−51^ including niobium oxides.^16,31,52,53^ In contrast to activation by pure metallic clusters, where carbonyl (CO) formation is observed, activation by metal oxides can lead to the formation of a carbonate radical anion moiety, [CO_3_]^−^.^30,54^

In previous work, we investigated CO_2_ activation on hydrated metal ions, showing that the number of attached water molecules can influence the degree of CO_2_ activation.^55,56^ In the current contribution, we show that a [CO_3_] moiety is formed when a CO_2_ molecule is adsorbed on [NbO_2_(OH)_2_]^−^. For this, we report gas-phase infrared multiple photon dissociation (IRMPD) spectra of [NbO_3_]^−^, of its hydration products [NbO_2_(OH)_2_]^−^ and [NbO_2_(OH)_2_]^−^(H_2_O), and of the monohydrated complex reacted with CO_2_, [NbO(OH)_2_(CO_3_)]^−^. The spectroscopy experiments utilize the free-electron laser for intracavity experiments (FELICE) free-electron laser in Nijmegen in the 240–2100 cm^–1^ spectral range covering metal-oxide as well as C–O and O–H bending vibrations and table-top tunable laser systems in our laboratory in Innsbruck in the 1200–4000 cm^–1^ region, providing access to the C–O and O–H stretch vibrations. The experiments are complemented with quantum chemical calculations of ion structure and spectra.

## Experimental and Theoretical Methods

Infrared multiple photon dissociation (IRMPD) spectra of the [NbO_3_]^−^, [NbO_2_(OH)_2_]^−^, and [NbO(OH)_2_(CO_3_)]^−^ anions were measured using the Fourier-transform ion cyclotron resonance (FT-ICR) mass spectrometer coupled to the FELICE beamlines at Radboud University in Nijmegen, The Netherlands. The ions are produced by laser vaporization of a solid niobium disk, followed by supersonic expansion in helium seeded with traces of O_2_, CO_2_, and H_2_O.^57−60^ The ions are accumulated in a rectilinear ion trap, which contains argon as a buffer gas, and then guided via ion optics to one of the four trapping/detection cells of a FT-ICR mass spectrometer, which is positioned inside the cavity of the free-electron laser.^14,61^ The spectral range studied in this work covers 240–2100 cm^–1^. Infrared spectra were recorded typically two or three times to ascertain reproducibility. The experimental spectra were measured in different ICR cells, corresponding to different distances from the laser focus and, consequentially, different photon fluxes.^14^ The first ICR cell is in the focus of the FELICE beam, while cell 4 is out of focus. The IR fluence is reduced by a factor of 2.3 between each neighbored cells.^14^

[NbO_3_]^−^, [NbO_2_(OH)_2_]^−^, [NbO_2_(OH)_2_]^−^(H_2_O), and [NbO(OH)_2_(CO_3_)]^−^ were also produced in Innsbruck, using a similar gas mixture and laser vaporization source. The ions were guided with an electrostatic lens system into the cell of a 4.7 T FT-ICR instrument, where they were irradiated using the tunable EKSPLA NT273 (1200–2210 cm^–1^) and EKSPLA NT277 (2250–4000 cm^–1^) laser systems with a maximum pulse energy of 50 and 500 μJ at 1 kHz, respectively.^62^ As the laser power for the EKSLPA systems is much lower than that of the free-electron laser, no fragmentation of [NbO_3_]^−^ and [NbO_2_(OH)_2_]^−^ was observed. Since IR absorption always competes with emission, the lower energy of the table-top OPO system is likely not sufficient to drive the IRMPD process. Therefore, [NbO_2_(OH)_2_]^−^ was tagged with an additional H_2_O molecule, and a spectrum of [NbO_2_(OH)_2_]^−^(H_2_O) in the range of 1250–4000 cm^–1^ was recorded. The spectrum of [NbO(OH)_2_(CO_3_)]^−^ could be measured in Innsbruck, Austria, without tagging.

All spectra were recorded at room temperature. The wavenumbers are calibrated using a calibration function determined by comparing the set wavelength to the output wavelength of the laser. In Nijmegen, the output wavelength was measured during the experiment, while in Innsbruck, the required data were recorded after the experiment.

The fragmentation yield *Y*_*F*_ for a given wavenumber is calculated as the logarithmic ratio of the summed intensities of the parent ion and all fragment ions (*I*_par_ and *I*_frag_, respectively) to the intensity of the parent ion:^63,64^
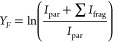


This yield is background corrected for black-body infrared radiative dissociation. In the Innsbruck experiments, this is done by subtracting the yield measured without laser light, and in the FELICE experiments, where no measurements without light are available, the lowest yield is taken as background.

Upon irradiation of [NbO_3_]^−^, there was no measurable fragmentation product but significant depletion of the parent ion signal, which we attribute to electron detachment. The spectrum for [NbO_3_]^−^ is therefore constructed from its wavelength-dependent depletion yield *Y*_*D*_, calculated as the negative logarithm of the intensity *I* of the [NbO_3_]^−^ signal at a given wavelength normalized to its maximum intensity *I*_0_:
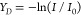
Here, too, a baseline correction was performed, except in the low-wavenumber range, where three very strong and broad absorption peaks cover the whole range, making the determination of a baseline difficult. After background correction, the respective yields are laser power corrected. For the final spectra, the yield of each range is normalized separately, and a five-point average is performed for smoothing. For comparison, spectra without corrections are shown in the Supporting Information (SI), Figure S1.

Based on extensive benchmarking calculations on the [NbO_3_]^−^ cluster, involving different methods (CCSD, CAM-B3LYP,^65^ BMK,^66^ ωB97XD,^67^ and MP2) and basis sets (def2TZVP, def2QZVP, aug-cc-pVDZ-PP, aug-cc-pVTZ-PP), we selected the B3LYP/aug-cc-pVTZ-PP combination for calculations of minimum structures and harmonic fundamental frequencies (Tables S1–S4). Wave function stabilization was performed on all structures prior to geometry optimization. Harmonic fundamental frequencies were scaled by a factor of 0.968. An anharmonic analysis of the vibrational modes was performed with VSCF and VPT2 methods, but all complexes except for [NbO_3_]^−^ showed nonphysical shifts for lower-lying frequencies (<600 cm^–1^). These calculations could therefore not be employed for the interpretation of the spectra. Instead, the frequencies of the overtones were estimated by doubling the fundamental frequency. All energies are zero-point-corrected except for the vertical electron detachment calculations. All structures shown represent local minima, the respective Cartesian coordinates are given in the SI along with further computational data. Because [NbO_3_]^−^ has a closed-shell electronic structure, we restricted our calculations to the singlet spin surface.^18^ The CCSD calculations for benchmarking and selected anharmonic vibration analysis calculations were performed with Molpro,^68^ and all others using the Gaussian software package.^69^

## Results and Discussion

**Figure 1:**
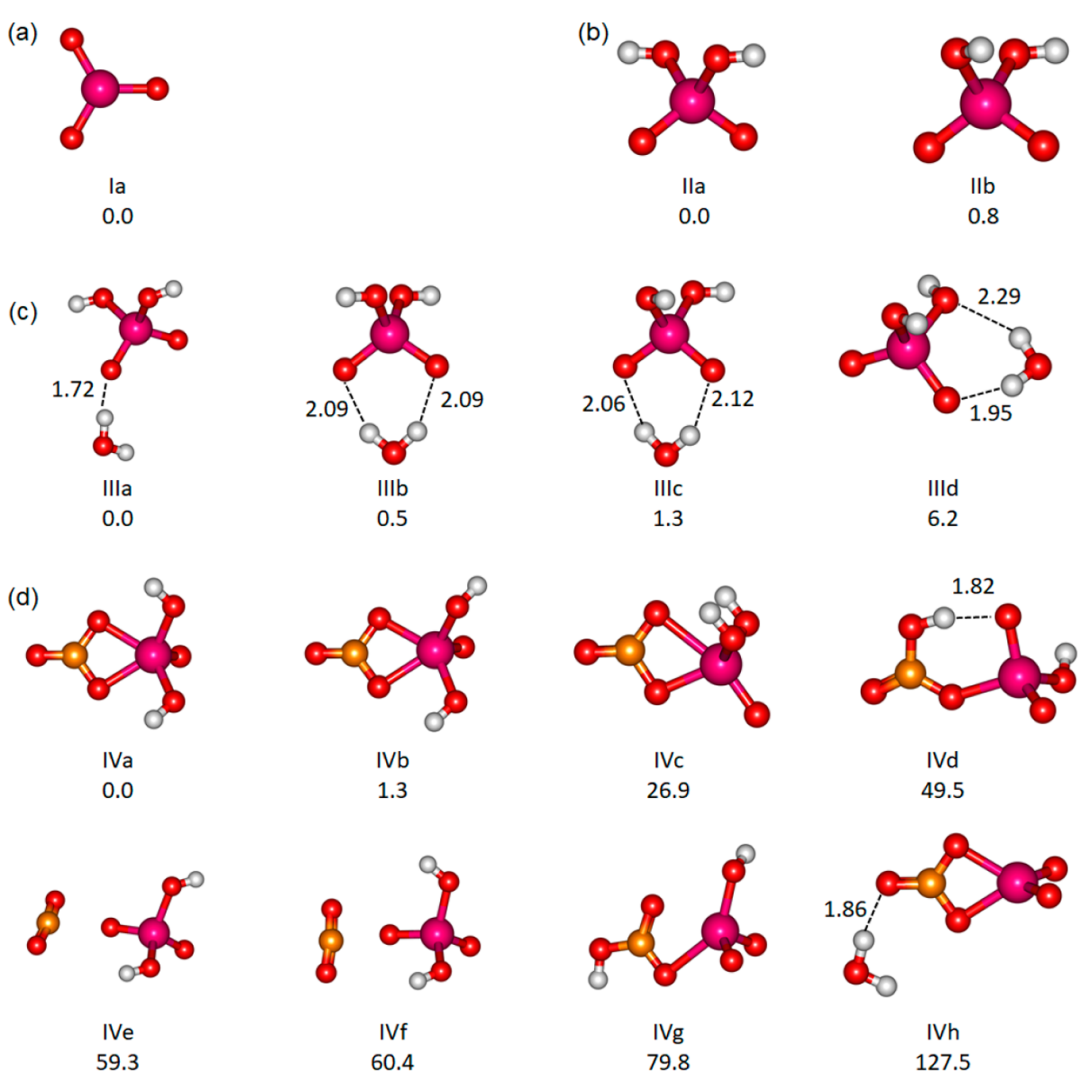
Isomeric structures of niobium species [NbO_3_]^−^ (a), [NbO_2_(OH)_2_]^−^ (b), [NbO_2_(OH)_2_]^−^(H_2_O) (c), and [NbO(OH)_2_(CO_3_)]^−^ (d) calculated at the B3LYP/aug-cc-pVTZ-PP level of theory. Relative energies are given in kJ mol^–1^. Hydrogen bond lengths are indicated in Å.

In this work, we experimentally and computationally investigated [NbO_3_]^−^, [NbO_2_(OH)_2_]^−^, [NbO_2_(OH)_2_]^−^(H_2_O) and [NbO(OH)_2_(CO_3_)]^−^ ions. Calculated energetically low-lying isomers of these species are shown in Figure 1 along with their relative energies in kJ mol^–1^. In the following, we will first discuss the measured spectrum for each species investigated, describe the computationally found isomers, and finally compare the associated calculated spectra with the experimental spectra.

### [NbO_3_]^−^

**Figure 2:**
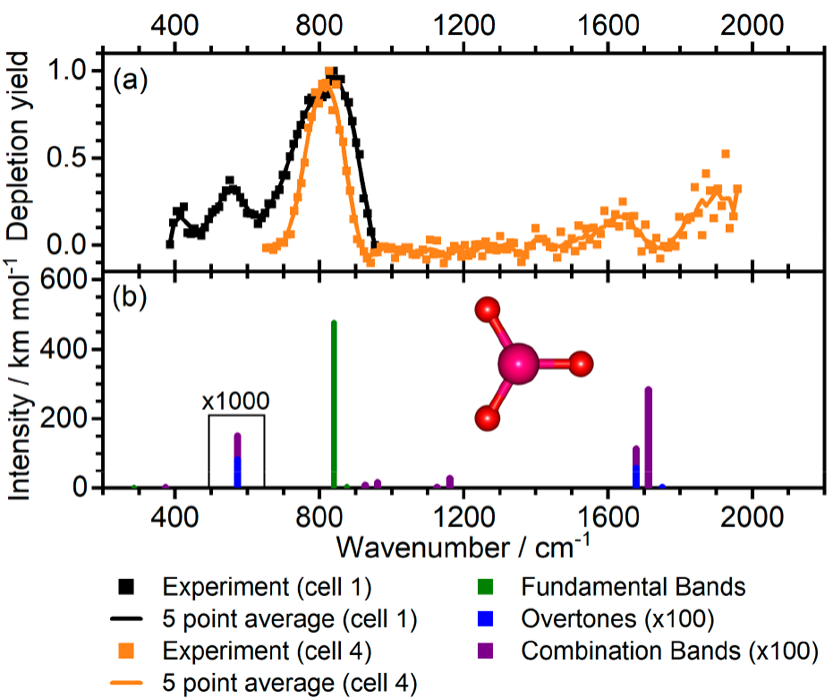
(a) Experimental IR depletion yield spectrum of [NbO_3_]^−^. The region from 400 to 1100 cm^–1^ was measured in cell 1 with two FELICE macropulses (black), while the region of 650–1960 cm^–1^ was measured in cell 4 with one macropulse (orange). (b) Spectrum calculated at the B3LYP/aug-cc-pVTZ-PP level using anharmonic analysis.

The experimental spectrum of [NbO_3_]^−^ is shown in Figure 2a, recorded using the FELICE setup. No fragmentation products but a reproducible, frequency-dependent loss of the parent ion was observed, which we attribute to electron detachment.^70,71^ As neither neutral molecules nor free electrons can be detected in the ICR cell, the depletion yield is presented in the spectrum.

The spectrum shows an intense band at around 800 cm^–1^ with a local maximum at around 840 cm^–1^. At this frequency, almost no parent ion is observed, and the width of the band suggests that it may be saturated. Two less intense bands are observed at 550 and 420 cm^–1^ and some very weak absorption features at 1600–1700 cm^–1^ that barely come out of the noise. Upon comparison of this spectrum to that taken using matrix isolation infrared spectroscopy, the 800 cm^–1^ band is likely the same as the matrix isolation band reported at 817.1 cm^–1^ assigned as the antisymmetric Nb–O stretch vibration of [NbO_3_]^−^.^22^

The [NbO_3_]^−^ has a closed-shell electronic configuration, and by optimizing its structure using B3LYP/aug-cc-pVTZ-PP, the molecule converged to a nearly planar structure of *C_3v_* symmetry with ONbO angles of 117.7° and the ONbOO dihedral angle of 150.4°. The planar structure of *D*_3*h*_ symmetry has the character of a transition state, lying 0.5 kJ mol^–1^ above the *C*_3*v*_ minimum, in an umbrella-style inversion well-known from the NH_3_ molecule.^18,23^ (At the CCSD/def2TZVP level, the planar configuration of *D*_3*h*_ symmetry is obtained as a minimum.) The umbrella motion lies at 98 cm^–1^ within the harmonic approximation; however, the frequency range below 200 cm^–1^ was not measured experimentally.

The experimental spectrum is compared with our frequency calculations in Figure 2b. The strong absorption band at ∼800 cm^–1^ matches the only fundamental mode calculated to have an appreciable IR intensity, the antisymmetric stretch of Nb–O, calculated at 840 cm^–1^, with potentially a weak contribution of the symmetric stretch vibration at 875 cm^–1^ of about 100 times lower intensity. The rotational envelope of the band at 300 K is calculated to be about 30 cm^–1^ fwhm assuming a laser line width of 10 cm^–1^ (Figure S2), while that of the experimental band is about 200 cm^–1^. This seems to confirm the suspected saturation broadening. The band around 550 cm^–1^ cannot be assigned to a fundamental, but its frequency matches that of the overtone of an ONbO bending vibration calculated at 573 cm^–1^ (fundamental: 286 cm^–1^) as well as a combination mode of both ONbO bending vibrations. Similarly, the band at 420 cm^–1^ could be attributed to one of the combination bands involving the umbrella mode and ONbO bending vibrations, although their calculated intensities are very low. An alternative assignment of this band could be to the antisymmetric stretch (maximum at 850 cm^–1^) due to the presence of <1% second harmonic of the FEL.^41^ Weak absorption features at 1600–1700 cm^–1^ are potentially assigned to overtones and combination bands of the Nb–O symmetric and antisymmetric stretching modes, but the frequencies do not match those of the calculations particularly well. Because these bands are very weak, this mismatch is deemed to be unimportant.

The calculated relative intensity of the bands is considerably different from that observed experimentally. As mentioned above, the 800 cm^–1^ band is likely saturated. Other factors that may be of relevance are: (a) errors in the quantum chemical calculations and (b) the fact that calculated intensities are linear absorption cross sections that cannot be compared directly to action spectroscopy yields, especially with the rather substantial energy of 435 kJ mol^–1^ needed for electron detachment, corresponding to the absorption of 45 photons at 800 cm^–1^ (Table 1). The spectrum of [NbO_3_]^−^ could not be measured in Innsbruck due to the lower laser power of the tabletop-level OPO system.

**Table 1:** Reaction Energies for Niobium Oxide Calculated at the B3LYP/aug-cc-pVTZ(PP) Level of Theory^a^

Reaction	Δ*E* (kJ mol^–1^)
[NbO_3_]^−^ + H_2_O → [NbO_2_(OH)_2_]^−^	–295
[NbO_2_(OH)_2_]^−^ + H_2_O → [NbO_2_(OH)_2_]^−^(H_2_O)	–51
[NbO_2_(OH)_2_]^−^ + CO_2_ → [NbO(OH)_2_(CO_3_)]^−^	–82
[NbO_3_]^−^ → [NbO_3_] + e^–^	435 (396)
[NbO_2_(OH)_2_]^−^ → [NbO_2_(OH)_2_] + e^–^	444 (401)
[NbO_2_(OH)_2_]^−^(H_2_O) → [NbO_2_(OH)_2_](H_2_O) + e^–^	494 (433)
[NbO(OH)_2_(CO_3_)]^−^ → [NbO(OH)_2_(CO_3_)] + e^–^	555 (489)

a For electron detachment energies, vertical values are given, and the adiabatic energies are included in parentheses.

### [NbO_2_(OH)_2_]^−^

**Figure 3:**
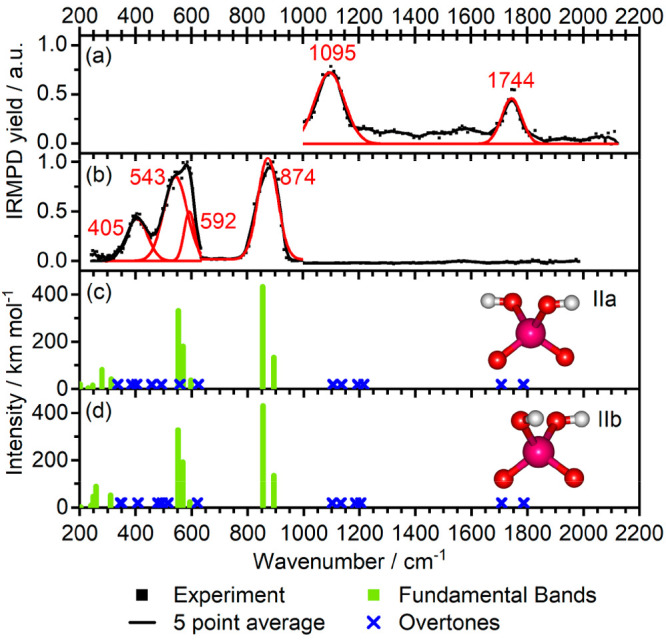
IRMPD spectra for [NbO_2_(OH)_2_]^−^ measured with full laser power in cell 1 (a) and reduced laser power in cell 4 (b). Calculated spectra within the harmonic analysis at the B3LYP/aug-cc-pVTZ-PP level are shown in panels (c) and (d). The harmonic fundamental and overtone frequencies are scaled by 0.968; position of overtones is indicated as crosses, as intensities were not calculated.

Irradiation of [NbO_2_(OH)_2_]^−^ with the intense FELICE laser resulted in the formation of [NbO_3_]^−^, while irradiation by the table-top OPO system did not result in fragmentation. Figure 3 shows IRMPD spectra of [NbO_2_(OH)_2_]^−^ recorded with FELICE under two different conditions: with maximum power (panel a) and with reduced power (panel b). At reduced power conditions, we observe three bands between 300 and 1000 cm^–1^, at 405, ∼550 and 874 cm^–1^, and no absorption features above 2000 cm^–1^. The 550 cm^–1^ band seems to consist of two bands, and when fitting with two Gaussian line shape functions, we find bands at 543 and 592 cm^–1^. These fitted peak positions and those of the other bands are indicated in Figure 3 in red. When irradiating under high power conditions (Figure 3a), two pronounced features appear at 1095 and 1744 cm^–1^ with some elevated background in between.

The calculations predict that the reaction of niobium oxide, [NbO_3_]^−^, with H_2_O produces niobium dioxydihydroxide, [NbO_2_(OH)_2_]^−^, consistent with what was previously reported by Sigsworth et al.^17^ Our DFT calculations show that the incoming water molecule coordinates to the metal center, enabling a proton transfer to an oxygen atom of [NbO_3_]^−^. In agreement with the results of Sambrano et al., who performed a full reaction pathway calculation considering singlet and triplet electronic states,^18^ our calculations indicate that the reaction is barrierless. The formation energy of [NbO_2_(OH)_2_]^−^ was calculated to be 295 kJ mol^–1^ (Table 1), and the back reaction that is driven by IR irradiation thus corresponds to the absorption of 31 photons at 800 cm^–1^. This high energy may be the reason why no fragmentation is observed with the table-top OPO system.

We have located two isomers of [NbO_2_(OH)_2_]^−^ with tetrahedral coordination of the metal center and an energy difference of 0.8 kJ mol^–1^ (Figure 1). Isomer IIa is the putative global minimum with *C*_2_ symmetry, while isomer IIb has *C*_*s*_ symmetry. The energy barrier for conversion from IIa to IIb was calculated as 2.8 kJ mol^–1^.

Calculated spectra of [NbO_2_(OH)_2_]^−^ for the two isomers IIa and IIb are shown in Figure 3c,d. The band at 872 cm^–1^ is attributed to the symmetric and antisymmetric stretch of the free Nb–O groups calculated at 854 and 893 cm^–1^ for both isomers. The experimental band structure centered at 543 and 592 cm^–1^ coincides with the Nb–OH symmetric and antisymmetric stretching modes (∼550–570 cm^–1^). The frequencies of the Nb–O stretch vibrations have blue-shifted from the values of the bare [NbO_3_]^−^ and the Nb–OH red-shifted, attributed to the weakening of the Nb–O bond upon protonation as electron density is withdrawn from the O–Nb bond upon formation of an O–H bond. Consequentially, the Nb atom’s electron density is distributed over the remaining Nb–O bonds, increasing the force constant. The 405 cm^–1^ experimental band is not well predicted by the calculations. It may correspond to Nb–O–H symmetric and antisymmetric twisting modes calculated to lie around 300 cm^–1^. We speculate that this mismatch is due to shortcomings in the harmonic calculations of low-energy vibrations (Experimental and Theoretical Methods). In the high-frequency range, the two clear bands only showed under high-power irradiation conditions. They are therefore unlikely due to fundamental modes. The band at 1095 cm^–1^ could correspond to overtones of Nb–OH stretching and OH bending modes, and the band at 1744 cm^–1^ to the overtones of Nb–O symmetric and antisymmetric stretching modes; see Figure 3c,d and Table S4. We can rule out an alternative assignment of the 1744 cm^–1^ band, as it is likely not due to the hydroxy OH stretch vibration excited by a fraction of the second harmonic of the FEL, as this mode is calculated at 3750 cm^–1^.

Based on the comparison between calculated and experimental spectra, we conclude the presence of one or both isomers. Their calculated spectra do not differ enough to allow for assignment of one or the other, and energetically they are expected to be both present, with the equilibrium constant calculated as 0.67 at 298.15 K using Gibbs energy at the B3LYP/aug-cc-pVTZ-PP level.

### [NbO_2_(OH)_2_]^−^(H_2_O)

The [NbO_2_(OH)_2_]^−^ ion itself could not be dissociated by the table-top lasers in Innsbruck. Therefore, it was complexed with an additional water molecule that could be eliminated by using the lasers. The spectrum of [NbO_2_(OH)_2_]^−^(H_2_O) was recorded in the range between 1250 and 4000 cm^–1^. In the lower wavenumber range, two peaks are observed at 1670 cm^–1^ and 2010 cm^–1^. The latter is reproducible, but barely above the noise level. In the high wavenumber range, there is one separate band at 3701 cm^–1^ and a broad structured band between 3000–3500 cm^–1^ with a maximum at 3192 cm^–1^, a clear sideband at 3023 cm^–1^ and a monotonously decreasing absorption ending around 3600 cm^–1^. The only fragmentation channel observed was the loss of one water molecule, confirming the stability of the [NbO_2_(OH)_2_]^−^ fragment ion.

The observation of a strong band around 1670 cm^–1^ appears to be consistent with the extra water molecule acting as a spectator, as this frequency is only slightly blue-shifted from the free water bending mode at 1595 cm^–1^.^72^ Bands in the 3600–3700 cm^–1^ region could either be due to free hydroxy OH stretch vibrations or due to OH groups of intact water molecules that are not coordinated to the [NbO_2_(OH)_2_]^−^ ion. The width of the structure below 3600 cm^–1^ is likely due to OH stretching vibrations involved in the hydrogen bonds.

The calculations indicate that [NbO_2_(OH)_2_]^−^ binds to a water molecule without activating it, leading to the formation of monohydrated [NbO_2_(OH)_2_]^−^(H_2_O) with a water binding energy of 51 kJ mol^–1^. The product has either one or two intermolecular hydrogen bonds between the water molecule and the oxygen atoms or the hydroxy groups of [NbO_2_(OH)_2_]^−^ (Figure 1). We found four isomers that differ in the orientation of the hydroxy groups and the position of the water molecule within an energy of 6 kJ mol^–1^ and several hydrogen bonding patterns. In the most stable isomer IIIa, one hydrogen bond is formed; isomers IIIb and IIIc have two hydrogen bonds between the water molecule and the free Nb–O groups.

All four isomers can coexist in the experiment due to their low differences in energy. The density of states calculated with the Beyer–Swinehart algorithm^73,74^ as a function of available energy for four isomers of [NbO_2_(OH)_2_]^−^(H_2_O) shows that isomer IIIa should be by far the most populated (Figure S3); however, the energy difference in isomer stability is close to the expected error in relative energy calculations.

**Figure 4:**
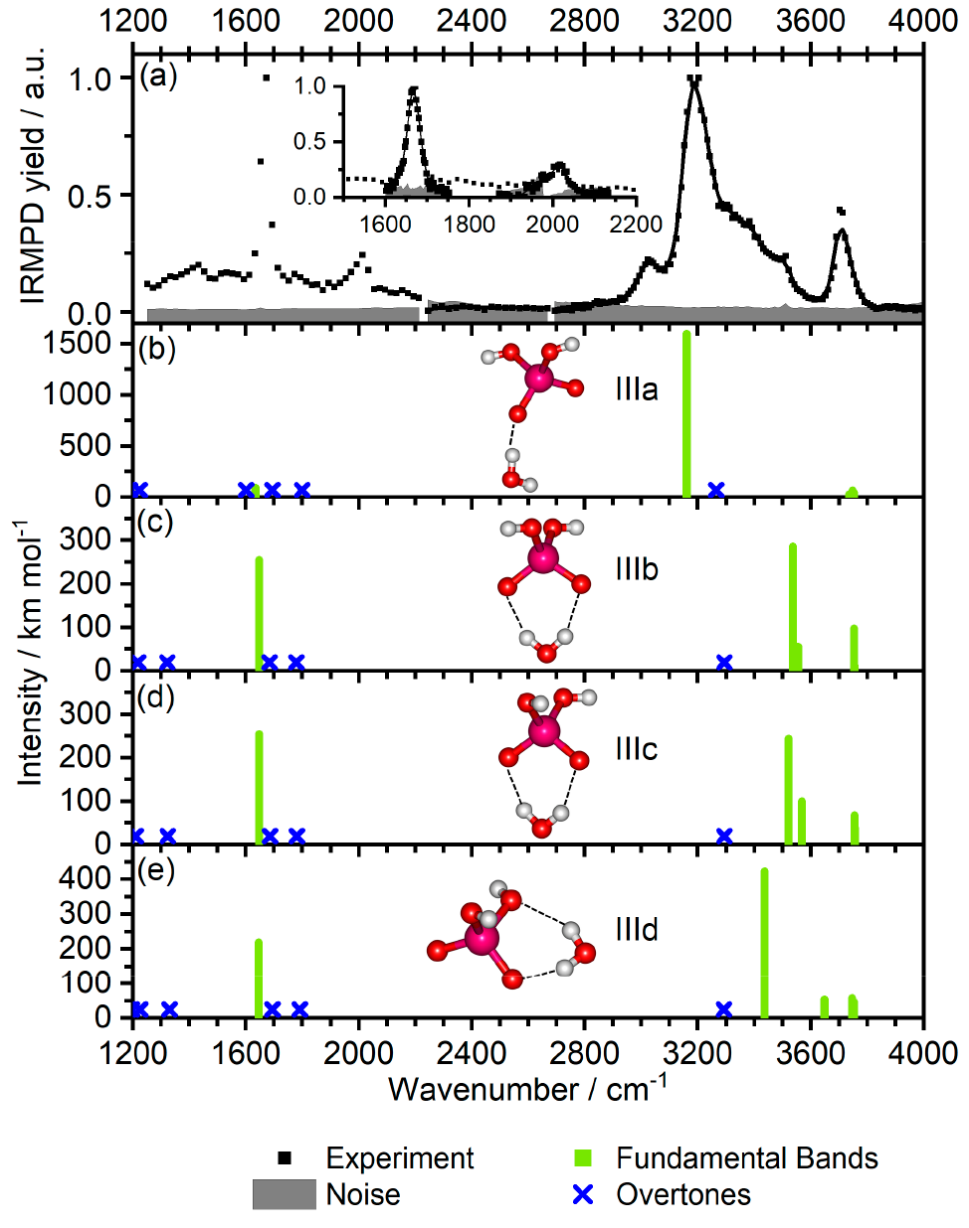
Experimental IRMPD spectrum of [NbO_2_(OH)_2_]^−^(H_2_O) (a); the inset shows measurements of the peaks with a higher number of points. Calculated IR spectra within harmonic analysis at the B3LYP/aug-cc-pVTZ-PP level for four most stable isomers are given in panels (b–e). The harmonic fundamental and overtones frequencies are scaled by 0.968; position of overtones is indicated as crosses as intensities were not calculated.

The experimental spectrum shown in Figure 4 is complemented by calculated spectra for the four lowest-lying isomers. The strong band at 1670 cm^–1^ is assigned to the water bending mode of the tagging water molecule. The maximum of the broad feature observed at 3192 cm^–1^ suggests the presence of isomer IIIa, exhibiting a “single acceptor” binding motif, which has a shorter hydrogen bond (1.72 Å) between the water molecule and one of the oxygen atoms of [NbO_2_(OH)_2_]^−^ compared with other isomers (Figure 1). The distance indicates a stronger hydrogen bond, leading to a withdrawal of electron density from bonding orbitals on the O–H bond, causing a redshift in the O–H stretch frequency. The experimental band at 3701 cm^–1^ is typical for free O–H stretching modes of hydroxy groups and is reproduced by the calculations for all isomers, being predicted to lie at 3754 cm^–1^ for isomer IIIb, while the simulated band is close to this position for the other isomers. This is different for the broad band between 3100–3500 cm^–1^ that can be explained by the presence of isomer IIIa with a single hydrogen bond. These modes correspond to symmetric and antisymmetric O–H stretching modes of the water molecule in a variety of hydrogen bonding arrangements. Such hydrogen bonding shifts the vibrational frequencies down from the values of the free water molecule, observed at 3657 and 3756 cm^–1^,^72^ and the rather mobile nature of the hydrogen bound water molecule leads to a drastic broadening of the band. Therefore, the broad band indicates that not only local minima but also other parts of the phase space are populated. This effect was shown previously for [CO_3_]^−^ water clusters employing molecular dynamics simulations, where a comparable water binding motif is present.^62^ This leaves two weaker bands for the assignment. First, the small band at 3023 cm^–1^. This could still be part of the broadened hydrogen bond affected OH stretch band or indicate the presence of further isomers. Finally, the small feature at 2010 cm^–1^ could possibly belong to the calculated combination bands of bending and rocking vibrational modes of the water molecule predominantly in the IIIa isomer.

The assignment of the observed spectrum of [NbO_2_(OH)_2_]^−^(H_2_O) to that of a niobium dioxydihydroxide complexed with a water molecule appears consistent with the assignment of the spectrum of [NbO_2_(OH)_2_]^−^ to a similar niobium dioxydihydroxide, partially by the verification of the free hydroxy OH stretch vibrational frequencies, which for the latter was calculated to lie at 3748 cm^–1^, and here is calculated to lie at 3754 cm^–1^ (isomer IIIa). The band is observed at 3701 cm^–1^, potentially shifted due to the interaction of the additional water molecule with the [NbO_2_(OH)_2_]^−^ structure.

### [NbO(OH)_2_(CO_3_)]^−^

**Figure 5:**
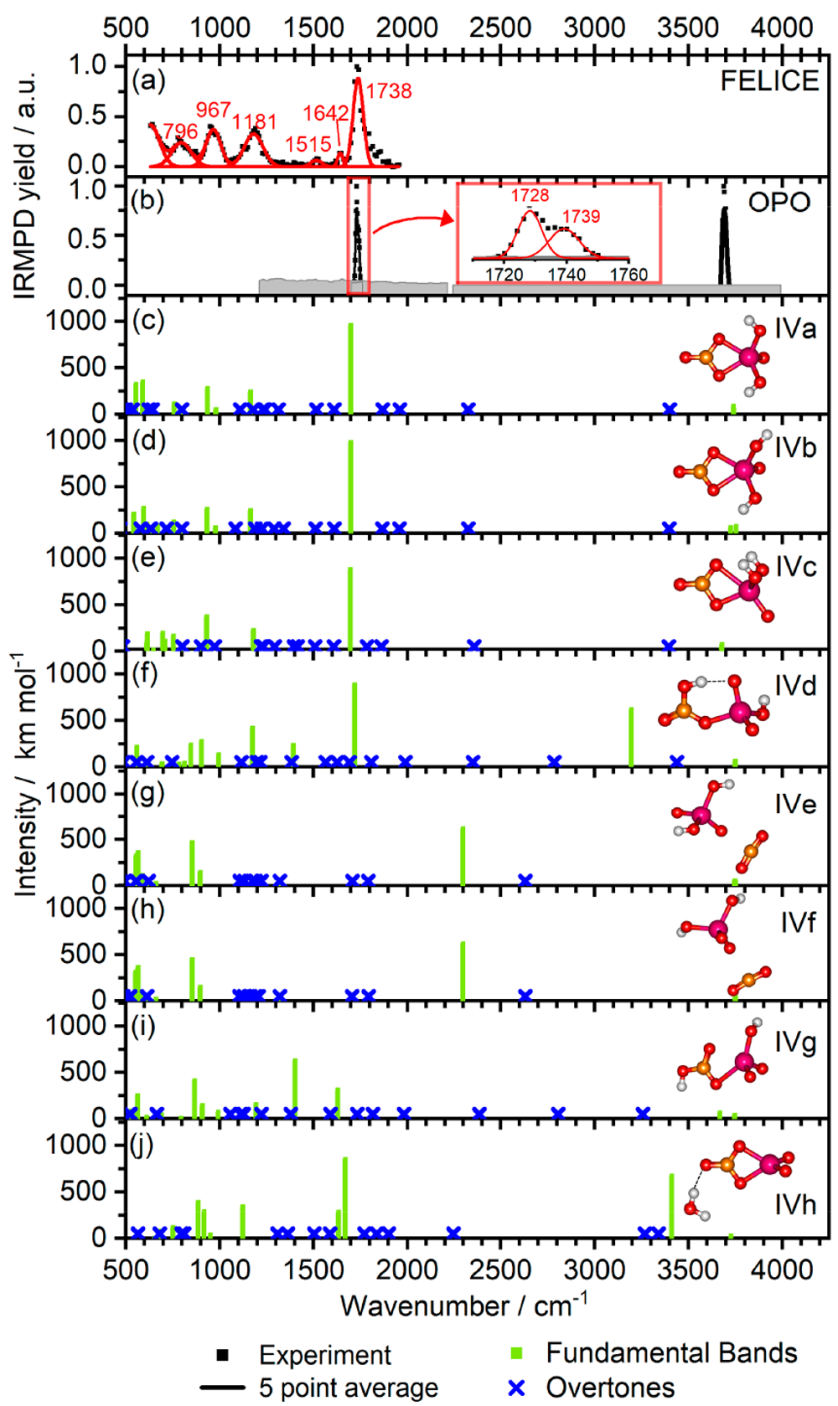
Experimental IRMPD spectra for [NbO(OH)_2_(CO_3_)]^−^ measured using the FEL in cell 1 (a) and the OPO (b) and calculated harmonic IR spectra at the B3LYP/aug-cc-pVTZ-PP level for different species (c–j). Red numbers indicate peak positions in cm^–1^. The harmonic fundamental and overtone frequencies are scaled by 0.968.

The experimental spectra of [NbO(OH)_2_(CO_3_)]^−^ were recorded in the 600–2000 cm^–1^ spectral range with the FEL in Nijmegen and in the 1210–4000 cm^–1^ range using the table-top lasers in Innsbruck. They are shown in Figure 5a,b. With FELICE, we observed CO_2_ loss as the primary loss channel, and for strongest bands, sequential loss of H_2_O. The spectrum shows relatively broad and probably saturated bands at 796, 967, 1181, and 1738 cm^–1^ and weak bands at 1515 cm^–1^ and 1642 cm^–1^. Figure 5b shows the spectrum measured using the table-top OPO system, where only CO_2_ loss was observed. Only two bands at 1730 and 3690 cm^–1^ are visible in the spectrum. The former has a shoulder to the blue, and fitting the band with two Gaussian line shape functions with a width of 12 cm^–1^ led to two maxima at 1728 and 1739 cm^–1^. Inspection of this band in the FEL spectrum (Figure 5a) shows that it has a tail to the blue, which appears consistent with the spectrally resolved band in the OPO spectrum.

**Figure 6:**
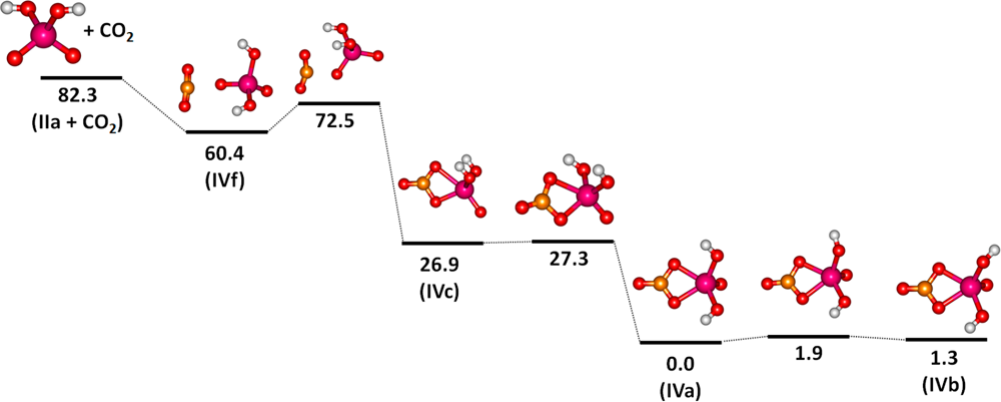
Potential pathway of CO_2_ activation on [NbO_2_(OH)_2_]^−^ as calculated at the B3LYP/aug-cc-pVTZ-PP level of theory. Energies are given in units of kJ mol^–1^.

According to calculations, CO_2_ binds via the O and C atoms in a η^2^-carbonato motif in isomers IVa, IVb, IVc, and IVh (with a CO_2_ binding energy of up to 82 kJ mol^–1^), while isomers IVd and IVg feature a bicarbonate structure. In isomers IVe and IVf, a weakly coordinating CO_2_ molecule is slightly deformed from its native linear geometry, indicating a very mild degree of activation. Our calculations, summarized in Figure 6, suggest that the activation energy of CO_2_ on [NbO_2_(OH)_2_]^−^ is 12.1 kJ mol^–1^ when starting with IVf with a weakly bound CO_2_ molecule, i.e., below the energy of the entrance channel. We predict that IVc is reached as an intermediate isomer that can be easily converted to IVa over a negligible barrier of 0.4 kJ mol^–1^.

In the three lowest-energy isomers, the newly formed [CO_3_] unit is bound to the niobium oxide via a bidentate motif, changing the coordination number of the niobium ion from 4 to 5. These three isomers differ only in the orientation and position of the hydroxy groups. In isomers IVd and IVg, the bicarbonate structure binds in a monodentate fashion, returning the coordination number of the niobium ion to 4. The high relative energy of isomers IVe, IVf, and IVh (see Figure 1 for energies) with weakly bound CO_2_ or H_2_O shows that the fully reacted [NbO(OH)_2_(CO_3_)]^−^ species will by far dominate the ion population under the room temperature conditions of our experiment.

The experimental spectra of [NbO(OH)_2_(CO_3_)]^−^ are complemented by calculated spectra for the eight lowest lying structures shown in Figure 5c–j. If the CO_2_ unit is only weakly activated, as in isomers IVe and IVf, the C=O asymmetric stretching vibration is predicted to lie at 2297 cm^–1^, close to its value for the free CO_2_ molecule observed at 2349 cm^–1^.^72^ On the other hand, if the CO_2_ is activated forming the [CO_3_] moiety, the frequency of this bound C=O stretching vibration red-shifts significantly to about 1700 cm^–1^. The observation of the intense band at 1738 cm^–1^ along with no sign of fragmentation around 2349 cm^–1^ is clear evidence of CO_2_ activation and [CO_3_] unit formation. Other features in the experimental spectrum can be explained by the very similar vibrational frequencies of the first three isomers. The band at 796 cm^–1^ could be assigned to the [CO_3_] scissoring mode predicted at 757 cm^–1^ and the out-of-plane bending mode at 806 cm^–1^, while the band at 967 cm^–1^ could be assigned to the Nb–O stretching predicted at 935 cm^–1^ (all values for isomer IVa, values for the other isomers are similar). The absorption band observed at 1181 cm^–1^ corresponds to the [CO_3_] antisymmetric stretching mode calculated at 1163 cm^–1^ for IVa and IVb and at 1180 cm^–1^ for IVc. The band observed at 3690 cm^–1^ is assigned to the combined O–H stretching mode of the hydroxy groups. The weak band at 1515 cm^–1^ could be assigned to combination bands of antisymmetric CO–Nb stretching and [CO_3_] stretching modes of isomers IVa and IVb, while the potential band at 1642 cm^–1^ coincides with the position of the overtone of [CO_3_] out-of-plane bending modes from isomers IVa, IVb, and IVc.

The absence of any activity near the 3195 cm^–1^ band predicted for the O–H stretching of the [HCO_3_] moiety of isomer IVd suggests that this isomer is not present in the experiment; analogously, the absence of a band corresponding to O–H stretching modes of the intact water molecule at 3411 cm^–1^ in isomer IVh rules out its presence. The exclusive presence of isomers IVa, IVb and IVc is consistent with their low relative energy.

The broad peak observed at 1738 cm^–1^ (Figure 5a) and the splitting observed in the OPO spectrum at 1728 and 1739 cm^–1^ (Figure 5b) correspond to the C=O stretching vibration, which is calculated at ∼1700 cm^–1^ for IVa, IVb, and IVc. The presence of the double band in the experiment would suggest the presence of isomer IVd, but this isomer is ruled out by the absence of an experimental band around 3200 cm^–1^. We speculate on the inaccuracy of the quantum chemical calculation, where the different orientation of the OH group in the three isomers mentioned above might slightly influence the position of the C=O vibration, also when dynamic effects are considered. Alternatively, the asymmetric structure of the peak might include hints toward a weaker Nb–O bond: As the Nb–O bond gets longer, the C=O bond grows stronger, and a blue-shift of the respective IR transition can be expected.

Given the thermodynamic stability, we expect that all IVa, IVb, and IVc isomers will be present in the experimental mixture. These observations are in line with the work by Knurr and Weber, who characterized species containing a [CO_3_] unit with similar frequency and binding motifs in [CoO(CO_2_)_*n*_]^−^ and [NiO(CO_2_)_*n*_]^−^.^48^ In larger clusters, like the Ti_3_O_6_^–^ model used by Asmis and co-workers, additional binding motifs, e.g., tridentate, become possible.^49^

## Conclusions

In this work, we presented IRMPD spectra of [NbO_3_]^−^, [NbO_2_(OH)_2_]^−^, [NbO_2_(OH)_2_]^−^(H_2_O), and [NbO(OH)_2_(CO_3_)]^−^. The spectrum for [NbO_3_]^−^ was measured only in Nijmegen, as the high laser power of the intracavity free electron laser experiment is necessary to efficiently excite the ion to achieve electron detachment, requiring about 45 photons at 800 cm^–1^, not taking into account radiative cooling of the hot ion. In addition to the strong, and likely saturated, fundamental absorption band associated with Nb–O stretching modes at 800 cm^–1^, which confirms the assignment of an earlier matrix isolation infrared spectroscopy study,^22^ we observe a relatively strong band at 550 cm^–1^ which we assign to an overtone.

When reacting [NbO_3_]^−^ with a water molecule, we experimentally confirm the results of Sambrano et al.^18^ and Sigsworth et al.,^17^ finding evidence for proton transfer from the water molecule to the [NbO_3_]^−^ forming niobium dioxydihydroxide. We computationally identified two isomers close in energy that cannot be distinguished within the current IRMPD spectrum, but they likely both contribute.

In contrast to the first water molecule, the uptake of a second water molecule does not lead to fragmentation but to the formation of [NbO_2_(OH)_2_]^−^(H_2_O), where the second water molecule forms two hydrogen bonds to the free oxygen atoms in niobium dioxydihydroxide. We found four different isomers that most likely all contribute to the experimental spectrum.

Finally, we observe the activation of CO_2_ on the [NbO_2_(OH)_2_]^−^ cluster, leading to the formation of a [CO_3_] unit. A comparison of the measured IRMPD spectra of [NbO(OH)_2_(CO_3_)]^−^ with different possible isomers provides clear evidence of the [CO_3_] moiety. The [CO_3_] unit is bound to the niobium atom in a bidentate fashion. The isomers with only weakly activated CO_2_ are too high in energy, and their spectral signatures are not observed.

Further investigation could help to reveal whether the bare [NbO_3_]^−^ is also able to activate CO_2_ or if the water molecule is necessary for the CO_2_ activation. Unfortunately, a pure [NbO_3_]^−^CO_2_ cluster was not observed in our experiments.

## Data Availability

The data underlying this study are available in the published article and its Supporting Information.

## Supporting Information Available

Uncorrected spectral data (Figure S1), rotational analysis of Nb–O antisymmetric stretch (Figure S2), density of states as a function of energy for [NbO_2_(OH)_2_]^−^(H_2_O) (Figure S3), tables with calculated frequencies (Tables S1–4), Cartesian coordinates (PDF)
Original experimental data shown in Figures 2–5 (XLSX)
