# Supplementary material for: Carbon Dioxide and Water Activation by Niobium Trioxide Anions in the Gas Phase
Source: J Phys Chem A. 2023 Apr 11;127(15):3402–11. doi: 10.1021/acs.jpca.3c01394 (PMC10123662; doi:10.1021/acs.jpca.3c01394)
Supplement: Supplementary file 1 — jp3c01394_si_001.pdf [file jp3c01394_si_001.pdf]

Supporting Information

Carbon Dioxide and Water Activation by Niobium Trioxide Anions in the Gas Phase

Magdalena Salzburger,[a] *Rizalina T. Saragi,*[a] *Frank J. Wensink,*[b] *Ethan M. Cunningham,*[a] *Martin K. Beyer,*[a] *Joost M. Bakker,*[b,*] *Milan Ončák,*[a,*] *and Christian van der Linde*[a,*]

a - Institut für Ionenphysik und Angewandte Physik, Universität Innsbruck, Technikerstraße 25, 6020 Innsbruck, Austria
b - Radboud University, Institute for Molecules and Materials, FELIX Laboratory, Toernooiveld 7, 6525 ED Nijmegen, The Netherlands

E-mail: j.bakker@ru.nl; milan.oncak@uibk.ac.at; christian.van-der-linde@uibk.ac.at

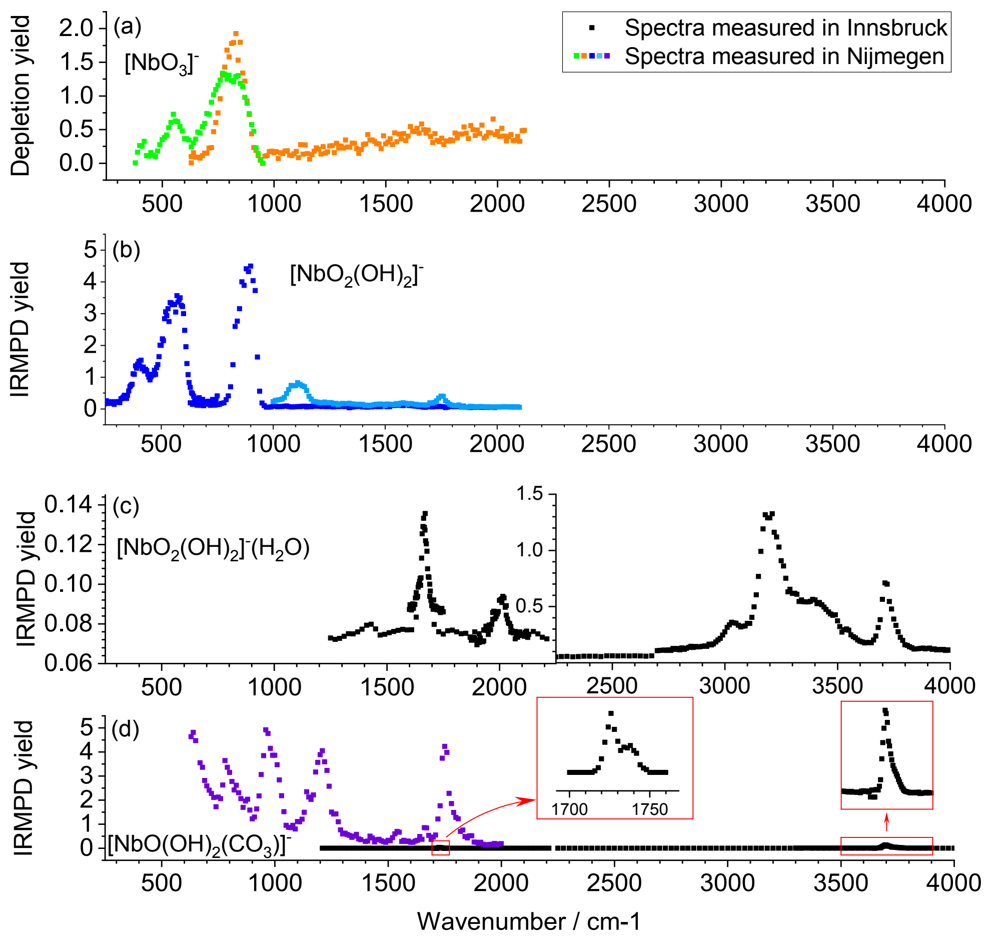

Figure S1. Uncorrected spectra of the measured clusters. a) Depletion of NbO_3^- without baseline correction and without correction for changes in laser power measured in cell 1 (green) or cell 4 (orange) , b-d) IRMPD yield of $[NbO_2(OH)_2]^-$, $[NbO_2(OH)_2]^-(H_2O)$, and $[NbO(OH)_2(CO_3)]^-$ without corrections for dissociation due to BIRD or CID and without correction for changes in laser power. Measurements of $NbO_2(OH)_2^-$ are recorded with high laser power (light blue) or low laser power (dark blue).

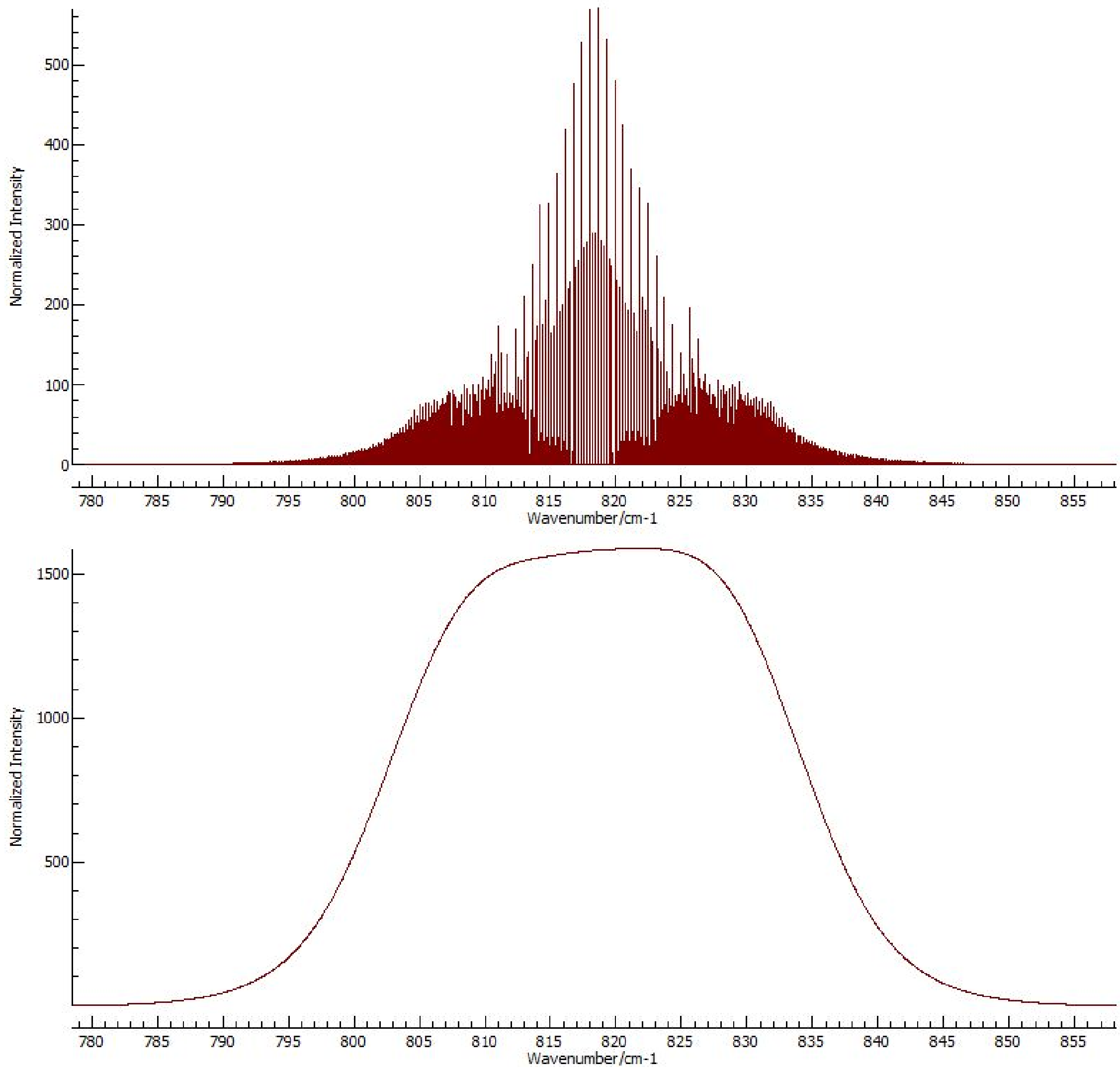

Figure S2. Simulated rotational envelope for Nb-O antisymmetric stretching on $[NbO_3]^-$ at 300 K, calculated by B3LYP/aug-cc-pVTZ-PP, assuming symmetric top. Simulation was done by PGOPHER program using no line broadening (upper) and Gaussian line-width of 10 cm^{-1} (lower).

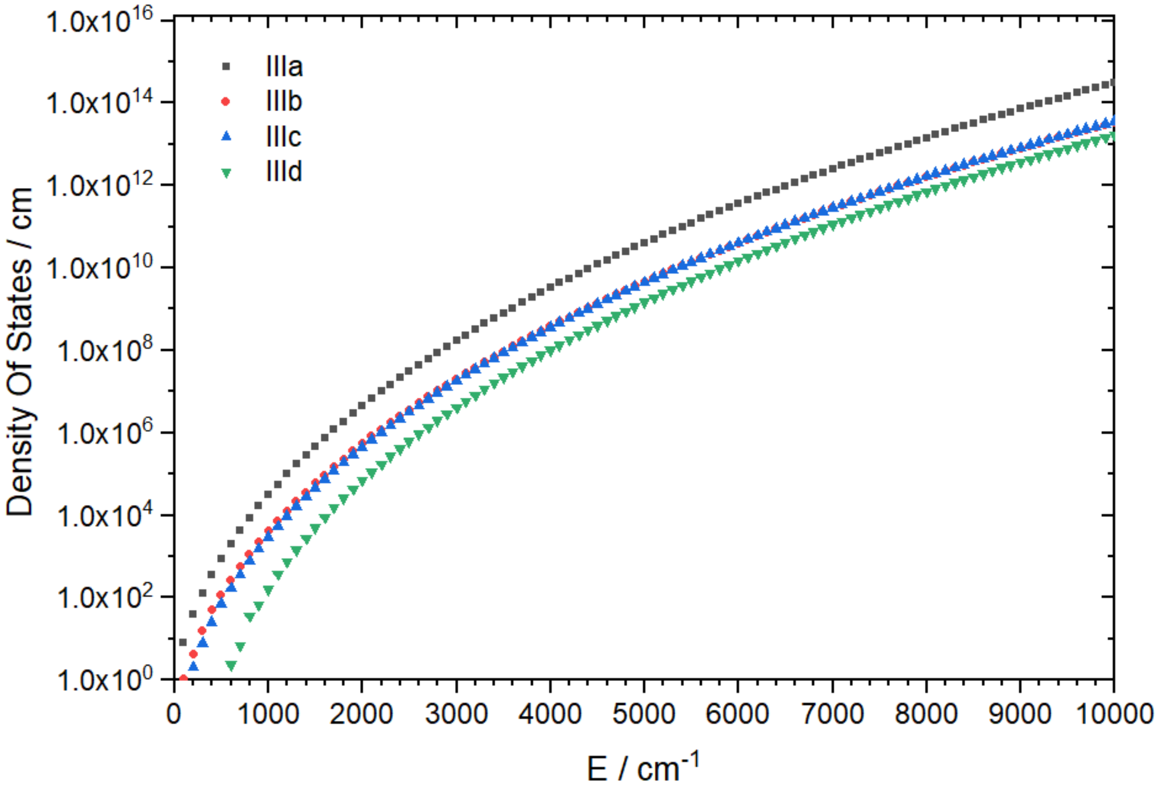

Figure S3. Density of states as a function of energy for $[NbO_2(OH)_2]^-(H_2O)$.

Table S1. List of harmonic frequencies (cm^{-1}, scaled) and their intensities (km mol^{-1}) of $[NbO_3]^-$ using different methods. The scaling factor used are 0.954 for CCSD, 0.963 for B3LYP and CAM-B3LYP, 0.955 for ωB97XD, 0.986 for BMK, and 0.952 for MP2. Def2-TZVP was used as the basis set.

CCSD		B3LYP		CAM-B3LYP		ωB97XD		BMK		MP2	
$\tilde{\nu}$	*I*	$\tilde{\nu}$	*I*	$\tilde{\nu}$	*I*	$\tilde{\nu}$	*I*	$\tilde{\nu}$	*I*	$\tilde{\nu}$	*I*
29.77	194.8	104.29	156.6	77.02	178.1	113.22	166.5	117.77	169.9	116.58	127.1
277.78	2.5	276.54	2.6	280.23	3.0	283.77	2.4	296.40	3.9	260.65	0.4
277.78	2.5	276.54	2.6	280.28	3.0	285.45	2.2	302.67	4.0	260.65	0.4
839.64	495.1	825.70	424.6	850.38	480.9	834.51	485.5	884.36	473.9	787.66	356.3
839.65	495.1	825.70	424.6	850.47	480.9	837.32	485.5	888.79	478.0	787.67	356.2
868.14	0.0	857.88	6.7	884.09	3.9	875.74	5.9	923.27	10.6	784.49	3.3

Table S2. List of frequencies (cm^{-1}, scaled) and their intensities (km mol^{-1}) of $[NbO_3]^-$ using B3LYP with different basis sets of def2TZVP, def2QZVP, and aug-cc-pVDZ-PP. The scaling factor of 0.968 was used for aug-cc-pVTZ-PP basis set, 0.963 for other basis sets.

def2TZVP		def2QZVP		aug-cc-pVDZ-PP		aug-cc-pVTZ-PP	
$\tilde{\nu}$	*I*	$\tilde{\nu}$	*I*	$\tilde{\nu}$	*I*	$\tilde{\nu}$	*I*
104.29	156.6	96.13	159.0	92.38	161.3	97.95	159.1
276.54	2.6	277.1	1.7	272.84	1.5	277.43	1.4
276.54	2.6	276.9	1.7	272.62	1.5	277.60	1.4
825.70	424.6	815.41	459	817.6	473.7	818.28	482.4
825.70	424.6	815.24	458.9	817.45	473.6	818.35	482.4
857.88	6.7	851.57	5.2	845.68	4.6	854.82	5.0

Table S3. List of harmonic and anharmonic frequencies for the overtones and combinations band (cm^{-1}) and their intensities (km mol^{-1}) of $[NbO_3]^-$ using B3LYP/aug-cc-pVTZ-PP. The harmonic frequencies are scaled by 0.968.

	$\tilde{\nu}_{harm}$	$\tilde{\nu}_{anharm}$	I
Overtones	195.90	156.14	4.46
	554.86	572.15	0.08
	555.20	572.62	0.08
	1636.57	1677.08	0.59
	1636.71	1677.43	0.58
	1709.64	1749.72	0.01
Combination bands	1673.17	1711.71	2.83
	1132.25	1161.58	0.26
	1095.78	1126.24	0.02
	952.77	960.96	0.15
	916.30	927.08	0.07
	375.38	372.68	0.01
	1673.10	1711.52	2.84
	1636.64	1678.23	1.14
	1095.71	1124.86	0.01
	916.23	926.84	0.07
	1132.42	1161.82	0.26
	1095.96	1125.31	0.01
	555.03	572.82	0.15
	375.55	372.96	0.01
	1095.89	1126.25	0.02

Table S4. Harmonic frequencies (cm^{-1}) and respective intensities (km mol^{-1}) of vibrations in isomer IIa and IIb $[NbO_2(OH)_2]^-$ using different methods employing the def2TZVP basis set. The harmonic frequencies are not scaled.

Method	Isomer IIa		Isomer IIb	
	$\tilde{\nu}$	I	$\tilde{\nu}$	I
B3LYP	3859.64	9.9	3859.44	31.1
	931.64	125.2	932.26	126.7
	622.04	58.6	618.88	42.0
	592.66	165.4	591.68	178.2
	327.08	52.0	326.04	71.1
	253.8	18.3	261.07	46.6
	202.83	26.0	182.25	0.5
	173.69	9.0	179.41	0.8
	3858.75	70.4	3857.96	44.7
	895.75	385.1	896.11	381.8
	634.77	0.3	629.08	0.0
	575.1	333.2	574.05	330.2

	295.3	94.6	268.49	107.9
	236.08	6.1	247.39	7.1
	209.07	26.0	211.07	2.6
CAM-B3LYP	3910.39	11.8	3910.87	40.0
	962.49	139.4	962.94	140.9
	614.62	20.7	615.10	32.2
	605.05	228.2	600.41	213.7
	333.07	56.2	332.65	77.3
	255.68	19.4	264.77	48.4
	207.95	26.6	185.27	0.7
	176.99	9.2	182.85	0.5
	3909.36	90.3	3909.46	57.2
	920.74	437.2	920.94	433.9
	635.48	25.1	630.19	33.6
	578.32	322.7	575.90	310.6
	300.07	106.7	270.85	127.3
	239.45	6.7	251.23	2.1
	212.44	29.7	214.35	2.1
BMK	3962.12	12.4	3961.94	44.1
	1006.44	144.5	996.95	155.8
	624.41	56.4	620.64	51.8
	545.14	209.5	544.08	208.5
	342.69	40.1	336.03	60.7
	269.47	21.9	266.82	58.5
	217.50	32.0	196.35	1.6
	186.97	9.6	182.27	1.3
	3961.09	94.8	3960.57	58.1
	972.42	452.2	959.54	440.1
	633.86	12.8	625.30	10.3
	516.67	353.3	516.96	349.0
	306.76	100.0	274.14	108.5
	257.91	3.2	264.02	13.2
	225.94	29.6	223.55	2.0
ωb97XD	3947.64	10.0	3947.40	32.8
	958.93	140.6	965.61	139.4
	617.44	47.4	616.17	16.7
	601.29	197.4	603.41	224.7
	333.12	51.8	335.35	68.7
	258.51	20.8	266.45	52.3
	207.45	27.0	187.89	1.6
	176.60	8.8	184.84	0.8
	3946.78	74.5	3946.15	47.5
	913.64	441.8	922.64	440.6
	633.54	3.2	629.94	8.4
	577.36	346.0	580.32	338.7
	300.86	101.8	274.29	119.9
	240.55	6.7	252.03	1.0
	212.63	27.0	216.49	2.5

MP2	3867.88	14.5	3867.52	37.4
	894.03	81.8	894.99	109.9
	608.04	37.8	606.60	26.3
	580.96	186.6	579.53	195.8
	319.17	43.8	319.89	64.5
	254.13	22.2	260.92	44.6
	199.32	28.5	179.34	0.4
	173.06	5.9	178.97	1.3
	3866.90	86.5	3866.30	59.2
	889.05	305.5	888.78	275.5
	626.00	2.5	621.16	3.0
	564.98	332.3	564.82	330.0
	290.53	90.9	264.37	92.8
	232.57	7.2	240.71	16.2
	202.81	22.7	205.46	3.2

List of Cartesian coordinates for all isomers (inÅng) along with zero-point corrected energies (in Hartree) as optimized at the B3LYP/aug-cc-pVTZ-PP level

```
CO2
E = -188.651729
C 0.000000 0.000000 0.000000
O 0.000000 0.000000 1.160464
O 0.000000 0.000000 -1.160464

H2O
E = -76.444957
O 0.000000 -0.000000 0.116988
H -0.000000 0.763513 -0.467950
H -0.000000 -0.763513 -0.467950

I
E = -282.764140
O 0.096369 -0.897596 1.534843
Nb 0.096535 0.029773 -0.000000
O -0.686970 1.642802 0.000000
O 0.096369 -0.897596 -1.534843

I, D3h
E = -282.763960
Nb 0.000000 0.000000 0.000000
O 0.000000 1.795863 -0.000000
O 1.555263 -0.897931 0.000000
O -1.555263 -0.897931 -0.000000

IIa
E = -359.321440
O 0.000000 1.455216 1.067215
Nb 0.000000 0.000000 0.068991
O 1.627371 -0.016364 -1.098904
O -0.000000 -1.455216 1.067215
O -1.627371 0.016364 -1.098904
H 2.109901 -0.842925 -1.160799
H -2.109901 0.842925 -1.160799

IIb
E = -359.321117
O -0.000331 1.515738 -1.015339
Nb -0.000006 0.073642 -0.000459
O 0.000390 0.501520 1.712514
O -1.629380 -1.024927 -0.391971
H 2.109274 -1.379400 0.358252
O 1.629287 -1.024829 -0.392507
H -2.108734 -1.379960 0.358974

TS between IIa and IIb
E = -359.320389
O 0.192449 -1.279318 -1.269257
Nb -0.000655 -0.064953 -0.004999
O -1.673029 0.993710 -0.303262
O -0.074356 -0.867272 1.564395
O 1.533420 1.221313 -0.041066
H -2.237972 1.152080 0.455421
H 2.436940 0.963533 0.143069

IIIa
E = -435.785988
O -0.478606 -0.395316 1.758063
Nb -0.452411 -0.004395 0.045899
O -1.493173 -1.383559 -0.942401
O -1.307839 1.770864 -0.239359
O 1.216407 0.048018 -0.567989
H -0.882859 2.389141 -0.837038
H -2.009783 -2.014681 -0.438112
O 3.890836 -0.036385 -0.140249
H 2.922240 -0.001579 -0.353354
H 3.898258 -0.221667 0.802116

IIIb
E = -435.785797
Nb -0.360945 0.000006 0.000070
O 0.671123 0.318627 1.398798
O 0.673383 -0.318245 -1.397215
O -1.520474 -1.582611 0.343688
H -1.537425 -2.295381 -0.297449
O -1.520568 1.582172 -0.345226
H -1.538560 2.295120 0.295679
O 3.269616 0.000005 -0.000380
H 2.643514 -0.162722 -0.725970
H 2.646570 0.163184 0.727547

IIIc
E = -435.785487
Nb 0.360105 -0.000005 -0.027399
O -0.720251 -0.000174 -1.426778
O -0.623931 -0.000019 1.439536
O 1.518588 -1.620563 -0.057255
H 1.578865 -2.170219 0.725941
O 1.518269 1.620778 -0.057373
H 1.578311 2.170593 0.725729
O -3.271169 -0.000028 0.049609
```

H -2.643064 -0.000022 0.789810
H -2.650481 -0.000117 -0.700023

IIId
E = -435.783609
O -0.669029 -0.837623 1.149401
Nb 0.432127 0.031509 0.063849
O 1.438793 -1.266081 -1.064234
O -0.714153 1.186494 -1.102993
O 1.563227 1.032019 0.963498
H -0.487612 2.112455 -1.206899
H 1.090087 -2.155367 -1.153503
H -2.783358 0.392613 -0.538215
O -3.247044 -0.245982 0.019281
H -2.510673 -0.592175 0.561172

IVa
E = -548.004513
C -1.991474 -0.000037 -0.027023
O -1.204963 -1.084294 0.006426
Nb 0.572782 -0.000023 0.084327
O 1.382499 -1.528250 -0.843581
O -1.204960 1.084246 0.006180
O 1.201851 -0.000133 1.691235
O 1.382325 1.528594 -0.843205
O -3.201407 -0.000036 -0.093310
H 0.811439 -2.116560 -1.343068
H 0.810580 2.116727 -1.342149

IVb
E = -548.004022
Nb -0.558554 0.022374 0.072157
O 1.260877 -1.055833 -0.045133
O -1.161847 0.269899 1.670693
O -1.266632 -1.718877 -0.492637
O -1.537616 1.205623 -1.148477
H -2.131375 1.878305 -0.809715
H -0.608179 -2.358244 -0.777989
C 2.009383 0.047293 -0.056766
O 3.218693 0.099910 -0.118267
O 1.184521 1.109135 0.005057

IVc
E = -547.994277
C -1.993705 -0.067549 -0.001028
O -1.071296 -1.117372 -0.009243
Nb 0.621177 -0.091926 -0.000837
O 1.023082 0.927685 -1.617148
O -1.363407 1.068326 0.007640
O 1.808403 -1.352275 -0.014701
O 1.021909 0.893277 1.637076
O -3.187307 -0.285530 -0.002579
H 0.320894 1.568334 -1.782444
H 0.322000 1.533030 1.814564

IVd
E = -547.985648
C -2.252893 0.226187 -0.028914
O -1.048565 0.730858 -0.274416
Nb 0.828685 0.011319 -0.076669
O 1.810319 0.917358 1.385680
O -2.275593 -1.077083 0.323257
O 1.733452 0.116525 -1.569768
O 0.451519 -1.674321 0.326567
O -3.277364 0.864468 -0.106412
H 1.748413 0.599648 2.288722
H -1.357291 -1.443281 0.348915

IVe
E = -547.981925
C 3.350488 -0.025258 0.033003
O 3.475801 -1.176471 -0.067819
O 3.399756 1.132074 0.141142
O -1.546071 1.858894 0.307126
Nb -0.963019 -0.013260 -0.062055
O -1.570604 -0.517745 -1.634455
O -1.728473 -1.210078 1.339230
O 0.808046 -0.082152 -0.009811
H -0.894164 2.467025 0.661242
H -2.432635 -1.807998 1.081658

IVf
E = -547.981497
O 1.296922 -0.000590 1.799999
Nb 0.962758 -0.000020 0.073652
O 1.772757 -1.623690 -0.759193
O -0.782738 0.000111 -0.241345
O 1.772729 1.624232 -0.758069
H 1.247849 -2.113721 -1.394877
H 1.247807 2.114778 -1.393341
C -3.320741 -0.000025 -0.027847
O -3.407790 1.159635 -0.024956
O -3.407418 -1.159708 -0.024489

IVg
E = -547.974111
C 1.959118 0.116098 -0.010942
O 1.425703 -1.022734 0.040288
Nb -0.695801 -0.090198 -0.007178
O -1.231301 -0.746193 1.529424
O 1.287035 1.170420 -0.072079
O -1.279416 -1.065482 -1.340803

O -1.638636 1.653127 -0.194579
O 3.314493 0.210834 -0.000563
H -1.889316 2.098271 0.617289
H 3.639424 -0.696522 0.049138

IVh
E = -547.955951
C 1.275762 0.723750 -0.000315
O 0.198573 1.522240 -0.000219
O 0.919129 -0.575798 -0.001169
O 2.427844 1.117306 0.000209
Nb -1.090202 -0.092091 -0.000159
O -2.029885 -0.414133 1.436461
O -2.034382 -0.414212 -1.433927
H 3.564777 -1.742449 -0.000714
O 4.240710 -1.058387 -0.000306
H 3.703016 -0.240433 0.000726
